# Eocene Western European endemic genus *Thaumastosaurus*: new insights into the question “Are the Ranidae known prior to the Oligocene?”

**DOI:** 10.7717/peerj.5511

**Published:** 2018-08-27

**Authors:** Davit Vasilyan

**Affiliations:** JURASSICA Museum, Porrentruy, Switzerland; Department of Geosciences, University of Fribourg, Fribourg, Switzerland

**Keywords:** *Thaumastosaurus*, MP16, Ranidae, Switzerland, Eocene, Grande Coupure

## Abstract

**Background:**

Amphibians, due to their ecophysiological peculiarities, have a physiology dependent on environmental conditions and sensitively respond to their changes. Here, the oldest record of the genus *Thaumastosaurus* is described, whose fossil record known exclusively from Western Europe is discussed in the scope of the climatic changes of 33.5–40.5 Ma.

**Results:**

In the present paper, the fossil remains of the genus from three localities in Switzerland (11 samples overall) have been studied and referred to the species *Thaumastosaurus bottii*. Its stratigraphic distribution has been revised and summarised. The studied localities present the stratigraphically oldest and the most eastern occurrences of the genus *Thaumastosaurus*. Eocene probable ranids (Ranidae indet./*Rana* sp./? *Rana* sp.) from Europe could be referred to *Thaumastosaurus*.

**Discussion:**

Their first occurrence of ranids most likely coincides with a warm phase of the global climate at 40 Ma, as tropical conditions were prevailing in Europe. As a result of the gradual cooling of the global climate, the tropical conditions in Europe were replaced by drier open habitats towards the latest Eocene at 34 Ma, when the latest occurrence of the European endemic genus *Thaumastosaurus* is known. Taking the fossil record and the climate evolution of that time into account, it can be concluded that *Thaumastosaurus* represents one of the groups among the vertebrates that disappeared during the large extinction event at the Eo–Oligocene transition, known as the Grande Coupure. The fossil finds of the genus from the studied localities allow to refer the previously suggested Eocene true frogs to the genus *Thaumastosaurus*, hereby stating the arrival of the true frog family Ranidae by the genus *Pelophylax* in Europe from the east at the earliest Oligocene.

## Introduction

**Figure 1:**
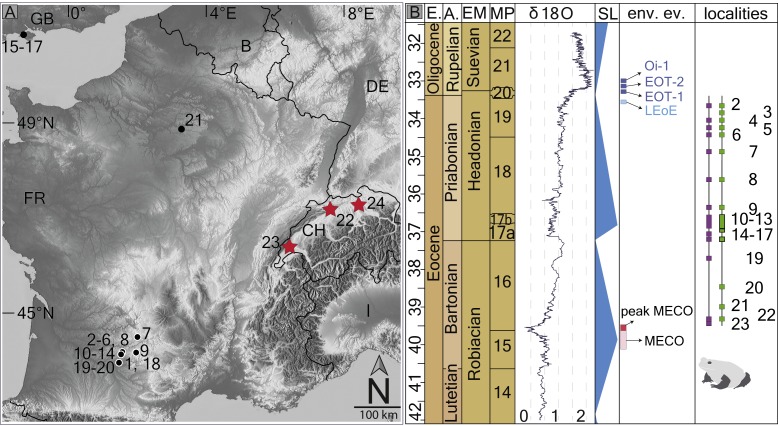
Fossil record of the genus *Thaumastosaurus*. (A) Geographic distribution of the localities (see below) with the *Thaumastosaurus* record (see more in Table S1). (B) Stratigraphic distribution of the localities indicated in (A), accompanied by curve of the *δ*^18^O development and sea level changes according to Vandenberghe, Hilgen & Speijer (2012), important environmental events (e.g., MECO, Oi–1) during the late Eocene and early Oligocene (Coxall & Wilson, 2011; Boscolo Galazzo et al., 2014), as well as the fossil record of the genus *Thaumastosaurus* and Eocene Ranidae/Rana sp./? Rana sp. (see more in Table S1). The heading abbreviation in (B) as follows: E, epoch; A, age; EM, European Land Mammal ages; MP, European Palaeogene Mammal Reference Level; SL, sea level; env. ev., environmental events; pk MECO/MECO, peak of/Mid–Eocene climatic optimum; LEoE, Late Eocene glaciation event; EOT–1, Eocene–Oligocene Transition event 1; EOT–2, Eocene–Oligocene Transition event 2, Oi–1, Oligocene 1 glacial maximum. The numbers correspond to the localities as following: 1, Quercy Phosphorite; 2, Escamps; 3, Rosières 1; 4, Coânac 1; 5, Rosières 2; 6, Sindou D; 7, Sainte–Néboule; 8, Monteils; 9, Cregols; 10, Perrière; 11, Malpérié; 12, Aubrelong 2; 13, La Bouffie; 14, Lebratières 1; 15, Southwest Headon Hill (=Headon Hill 3); 16, Rodent bed, Hordle Cliff locality; 17, Mammal bed, Hordle Cliff locality; 18, Quercy Phosphorite; 19, Le Bretou; 20, Lavergne; 21, Grisolles; 22, Verrerie de Roches; 23, Les Alleveys; 24, Dielsdorf. The localities 1 and 18 Quercy Phosphorite as well as 24, Dielsdorf (Table S1) are not included into the row “taxa occurrences” due to the age inaccuracy. Map data © OpenStreetMap contributors, CC BY-SA.

Climate changes strongly shape the spatial distribution and diversity of animals (Winter et al., 2016). In the geological past, numerous examples exist for the climatic and environmental impacts (Zachos, Dickens & Zeebe, 2008) on animal distributions, especially ectothermic vertebrates (e.g., amphibians and reptiles) (Böhme, 2003; Rage, 2012). Sharp climatic changes have been documented as affecting the entire vertebrate associations in the terminal Eocene—e.g., the Grande Coupure (Legendre, 1989). During the late middle–late Eocene (MP16–20), anuran fauna in Europe were represented by several clades: Alytidae (earlier Discoglossidae), Pelodytidae, Pelobatidae, ?Ranidae (cf. *Rana* sp.), Palaeobatrachidae, and *Thaumastosaurus* (Rage & Ford, 1980; Rage, 2012). Among them, *Thaumastosaurus* and cf. *Rana* sp. can be considered as the most abundant taxa in the European fossil record (Rage, 1984; Laloy et al., 2013). The cf. *Rana* sp./Ranidae indet. and the European endemic genus *Thaumastosaurus* co-occur in the stratigraphic record. So far, their stratigraphic record begins in MP16 (Le Bretou, France) and continues until MP19 (Escamps, France), perhaps MP20. It is important to note that no amphibians and reptiles are known from MP15 (Rage, 2012), and the presence/absence of *Thaumastosaurus* and Ranidae from this time cannot be confidently stated. In the terminal Eocene, the genus *Thaumastosaurus* disappeared at the “Grand Coupure” event (Rage, 2012) (Fig. 1A; Table S1), when all herpetofauna (mammalian as well) declined and were gradually replaced by fewer fauna. These changes are best documented for the amphibian and reptile fauna of the Quercy region (Rage, 2012) (Fig. 1C).

The fossil record of genus *Thaumastosaurus* includes four species: *Thaumastosaurus gezei* Rage & Roček (2007) from the Quercy Phosphorites, France (MP16–19/20); *Thaumastosaurus bottii* De Stefano (1903) from localities in France (MP16–20) (Roček & Lamaud, 1995); and *Thaumastosaurus sulcatus* and *Thaumastosaurus wardi* from several localities in England (MP17) (Holman & Harrison, 2002; Holman & Harrison, 2003) (see Table S1 for the detailed list). In the fossil record, the genus is known from articulated partial or complete skulls, as well as disarticulated elements of the skull and postcranial skeleton. A recent study (Laloy et al., 2013) showed that the mummy frog *Rana plicata* from the Quercy Phosphorite (MP17?) belongs to the species *T. gezei*. The therein (Laloy et al., 2013) provided phylogenetic analysis, based on the morphological characteristics of the partial skeleton (complete skull, vertebral column, and pectoral girdle) of the mummy specimen, nested the genus *Thaumastosaurus* within the clade Natatanura (Ranoides, Anura) that includes recent genera living in Africa. Hence, an African origin for the European Eocene genus *Thaumastosaurus* has been reassessed (Laloy et al., 2013).

Whereas the fossil material of the genus *Thaumastosaurus* is represented by cranial and partial postcranial skeleton, the other two frog taxa assigned to cf. *Rana* sp./Ranidae indet./Ranoidea indet. are only known from the same time period by postcranial material, including a few elements of the vertebral column, pectoral and pelvic girdles, as well as limb bones (Table S1) (Rage, 1984; Holman & Harrison, 1999). The cf. Rana sp./Ranidae indet. is considered to be the oldest ranid of the European continent, which possibly belongs to the extant genus *Rana* s.l. (Rage, 1984). The palaeogeographic relationships of this taxon are still unclear, but it is clearly an immigrant group (Rage & Augé, 2003).

**Figure 2:**
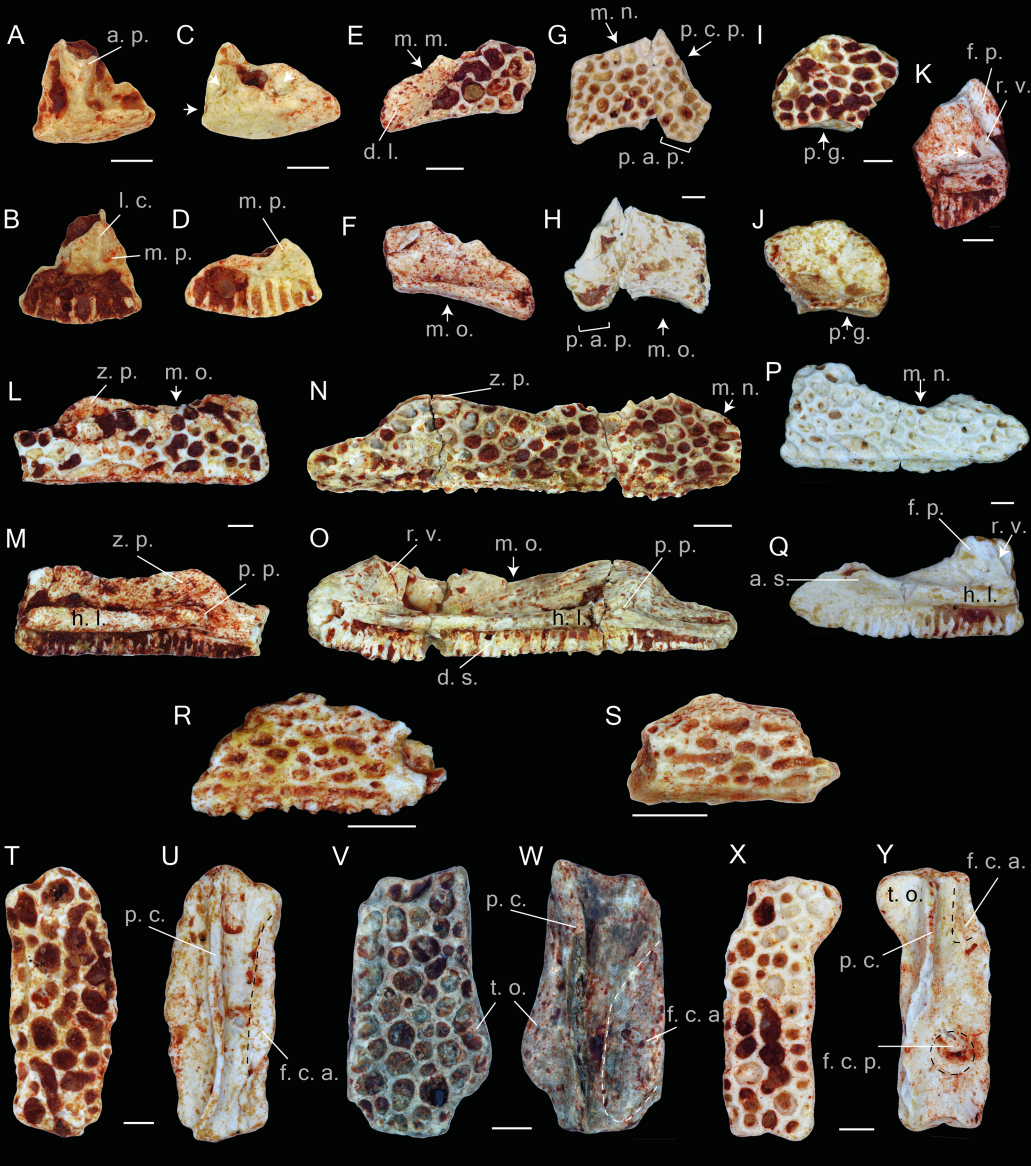
Praemaxillae, maxillae, nasals, squamosals and frontals of *Thaumastosaurus bottii*. (A–D) left premaxillae, MJSN VRR006-575 (A–B) and -296 (C–D), loc. V_p24; (E–F) left nasal, NHMB V.R.149, loc. V_K72; (G–H) right nasal, NHMB V.R.243, loc. V_06; (I–J) right nasal, MJSN VRR006-596, loc. V_p24; (K–S) maxillae, NHMB V.R.245 (K), V.R.246 (L–M), V.R.247 (N–O), V.R.248 (P–Q), loc. V_06, MGL 101581 (R), 101582 (S), loc. LA_86; (T–Y) right frontoparietals, NHMB V.R.239 (T–U), V.R.240 (V–W), V.R.241 (X–Y), loc. V_06; (A, C, E, G, I, T, V, X) dorsal; (B, D, F, H, I, U, W, Y) ventral; (K, L, N, P) lateral; (M, O, Q) medial views. Abbreviations: a. p., alary process; a. s., anterior spine; d. l., groove for ductus nasolacrimalis; d. s., dental shelf; f. c. a., facies cerebralis anterior; f. c. p., facies cerebralis posterior; f. p., frontal process; h. l., horizontal lamina; l. c., longitudinal crest; m. m., margo maxillaris; m. n., margo nasalis; m. o., margo orbitalis; m. p., medial process; p. a. p., paraorbital process; p. c., pars contacta; p. c. p., parachoanal process; p. g., posterior groove; p. p., pterygoid process; r. v., recessus vaginiformes; t. o., tectum supraorbitalis; z. p., zygomatic process; arrows indicate foramina or fossae. The dashed lines in U, W, Y indicate the outlines of the facies cerebralis. Scale bars equal 1 mm.

The objectives of the present study are: (1) to describe new material of *Thaumastosaurus* from three Swiss localities (Fig. 1B); (2) to revise the stratigraphic records of the genus and of the cf. *Rana* sp./Ranidae indet./Ranoidea indet. from the Eocene of Europe; and (3) to analyse climatic influence on their spatial and temporal distribution.

### Geological settings

All fossil localities are presented by either a single or network of karstic fillings in the Jurassic limestones of the Jura Mountains. The pockets are filled with red clays, sometimes alternated with sands, sandy layers and/or ferruginous pisoliths (“Bonhnerz–pellets”). The ages of the karstic pockets are dated to the Paleogene Mammalian zones using the mammalian association(s). Below, short descriptions of the pockets and a list of small mammal species important for the biochronology are given.

#### La Verrerie de Roches, Canton Jura

The localities of La Verrerie de Roches represent a network of several fissure fillings (pockets) of different shapes and orientations. Among the localities of La Verrerie de Roches, the pocket nr. 24 provides the largest part of the studied material and contains a small mammal assemblage. Based on the species composition of the latter, the fauna correlates to the Robiacian European Land Mammal Age–MP16, corresponding to the Bartonian stage, late middle Eocene (Becker, Rauber & Scherler, 2013). A latest study on primate fauna from the pockets of the La Verrerie de Roches suggested a slightly older age of transition of the MP15–MP16 zone (Minwer-Barakat et al., 2017). The ages of other karstic pockets (see below) cannot be directly dated due to the lack of any mammal taxa. An age similar to pocket nr. 24 can be supposed for the remaining pockets based on: (1) the very close locations of the pockets and by the presence of connections among pockets by small channels; (2) the deposition in the same reddish clay; (3) the same preservation of fossil bones; and (4) the same amphibians and reptile taxa (work in progress).

*Mammal species*. MP16 *Elfomys engesseri*, *Elfomys* cf. *tobieni*, *Mixtotherium lavergnensis*, *Paradelomys crusafonti, Paradelomys ruetimeyeri, Sciuroides* cf. *romani*, (Becker, Rauber & Scherler, 2013), between MP15 and MP16 *Necrolemus* aff. *anadoni*, *Pseudoloris parvulus*, *Pseudoloris pyrenaicus* (Minwer-Barakat et al., 2017).

#### Les Alleveys, Canton Vaud

The karstic pocket of the Les Alleveys is located in the quarry of the same name. It represents a horizontal karstic fissure. The age of the pocket is estimated as basal MP16 using the small mammal assemblage. The Les Alleveys small mammal fauna is slightly older than that of the La Verrerie de Roches pocket nr. 24 (Hooker & Weidmann, 2000; Hooker & Weidmann, 2007; Becker, Rauber & Scherler, 2013).

*Mammal species. Elfomys engesseri*, *Leptolophus stehlini*, *Palaeotherium castrense castrense*, *Palaeotherium lautricense, Sciuroides siderolithicus* (Hooker & Weidmann, 2007).

#### Dielsdorf, Canton Zürich

The karstic pockets are located in the Baden and Wetting limestone quarry. The pockets are vertically oriented in the limestone. The ages of the pockets are not precisely identified due to the lack of mammal species important for the dating. The ages range from MP14–MP20 for Fissure 1; MP16–MP19 for Fissure 2; MP16–MP20 for Fissure A; and an unknown age for Fissure? (Rosselet, 1991; Rosselet, 1993).

*Mammal species.* Fissure 1: *Lophiotherium* cf. *siderolithicum* (*Lophiotherium* cf. *robiencense*), *Necrolemur* cf. *antiquus*, “*Adelomys*” cf. *vaillanti*, *Anchilophus* cf. *dumasi*, *Amphiperatherium bastbergense*; Fissure 2: *Lophiotherium* cf. *cervulum*, *Suevosciurus minimus*, *Simaphicyon helveticus*; Fissure A: *Lophiotherium* cf. *cervulum*, *Anoplotherium laurillardi*, *Choeromorus* cf. *helveticus*, *Simamphicyon* ? *helveticus*, *Suevosciurus* cf. *minimus* (Rosselet, 1991; Rosselet, 1993).

## Material & Methods

The disarticulated fossil materials from ten karstic pockets (samples) in La Verrerie de Roches, three different samples from the Les Alleveys karstic pocket and fossil bones from four karstic pockets from the Dielsdorf locality have been studied. Aside from anurans, the herpetofaunal assemblage of the studied localities also contains caudates, squamates and crocodiles. They will be published separately.

Below, the localities where the fossil materials are stored and the samples, with their abbreviated names (in brackets) and acronyms of the collections/museum, are listed. The abbreviated sample or pocket names are used in the listing of the studied material in the following text as well as in the figure captures.

List of the studied karstic fillings with the abbreviated names and depositories:

1. La Verrerie de Roches, Stehlin collection (V_St), NHMB.
2. La Verrerie de Roches, 10.10.71 (V_71), NHMB.
3. La Verrerie de Roches, Karfreitag 1972 (V_K72), NHMB.
4. La Verrerie de Roches, 15.7.81 (V_81), NHMB.
5. La Verrerie de Roche, 2006 (V_06), NHMB.
6. La Verrerie de Roche, pocket 18 (V_p18), MJSN.
7. La Verrerie de Roche, pocket 20 (V_p20), MJSN.
8. La Verrerie de Roche, pocket 22 (V_p22), MJSN.
9. La Verrerie de Roche, pocket 24 (V_p24), MJSN.
10. La Verrerie de Roche, pocket 29 (V_p29), MJSN.
11. Les Alleveys, 1986 (LA_86), MGL.
12. Les Alleveys, 1991 (LA_91), MGL.
13. Les Alleveys, coll. Chavannes, 1983 (LA_83), MGL.
14. Dielsdorf Spalte 1 (D_1), PIMUZ.
15. Dielsdorf Spalte 2 (D_2), PIMUZ
16. Dielsdorf Spalte A (D_A), PIMUZ.
17. Dielsdorf Spalte? [Unknown] (D_?), PIMUZ.

## Results

### Systematic palaeontology

**Table:** 

**Figure 3:**
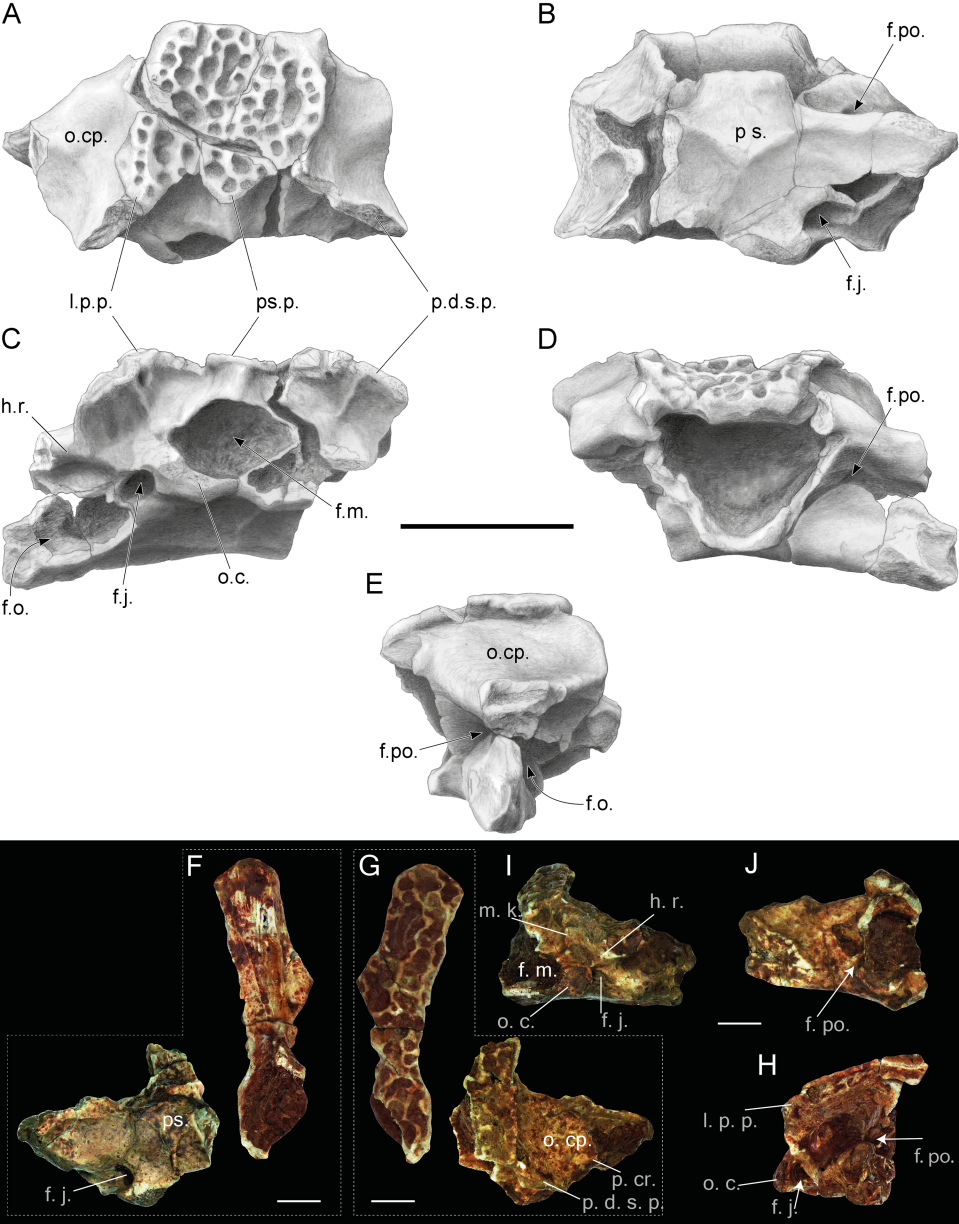
Incomplete skulls with prooticooccipital, frontoparietal and parasphenoid bones of *Thaumastosaurus bottii*. (A–E) NHMB V.R.31, loc. V_St; (F–H) NHMB V.R.37, loc. V_St. The bones are figured in (A, G) dorsal; (B, F) ventral; (E, H) lateral; (C, I) posterior; (D, J) anterior views. Abbreviations: f. j., foramen jugulare; f. m., foramen magnum; f. o., fenestra ovalis; f. po., foramen prooticum; h. r., horizontal ridge; l. p. p., lateral paraoccipital process; o. c., occipital condyle; o. cp., otic capsule; p. cr., parotic crista; p. d. s. p., prominentia ducti semicircularis posterior; ps., parasphenoid; ps. p., posterior superior process. Scale bar equals 5 mm (A–E) and 2.5 mm (F–J).

**Figure 4:**
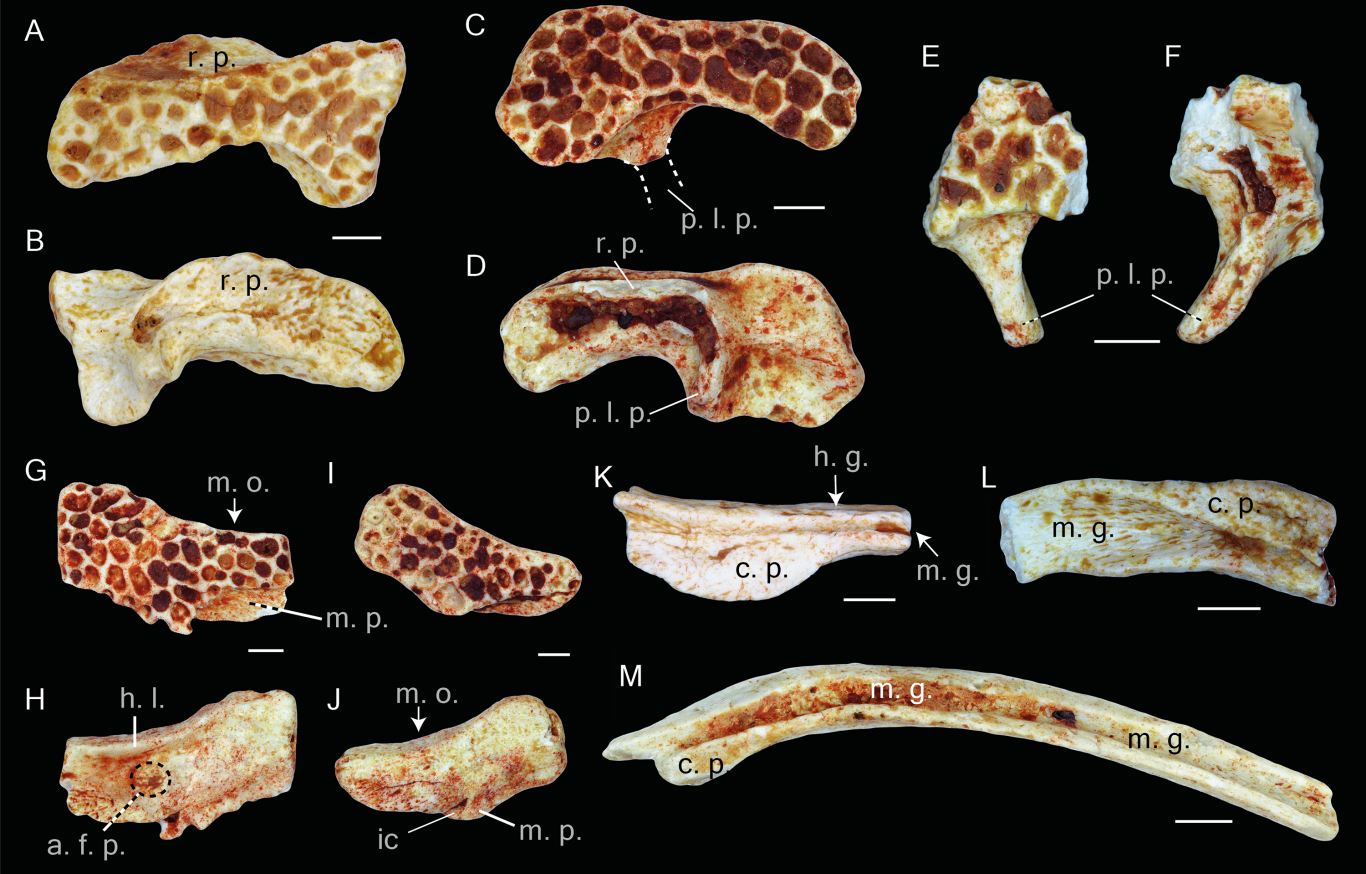
Squamosal and prearticulars of *Thaumastosaurus bottii*. Posterior parts of (A–B) one left; NHMB V.R.145, loc. V_K72, and (C–F) two right squamosals; MJSN VRR006-589 (C–D), loc. V_p24 and NHMB V.R.221 (E–F), loc. V_06 correspondingly. Anterior portions of two left squamosals (G–J) MJSN VRR006-591 (G–H) and VRR006-590 (I–J), loc. V_p24; (K) one right; MJSN VRR006-611 and (L, M) two left angulars; MJSN VRR006-613 (L) and MJSN VRR006-612 (M), loc. V_p24 correspondingly. The bones are figured in (A, C, E, G, I) lateral; (B, D, F, H, J) medial; (K–M) dorsal views. Abbreviations: a. f. p., articulation facet with pterygoid; c. p., coronoid process; ic, incrisura; h. g., horizontal groove; h. l., horizontal lamina; m. g., Meckelian groove; m. o., margo orbitalis; m. p., maxillary process; p. l. p., posterolateral process; r. p., ramus paroticus. Scale bars equal 1 mm.

**Figure 5:**
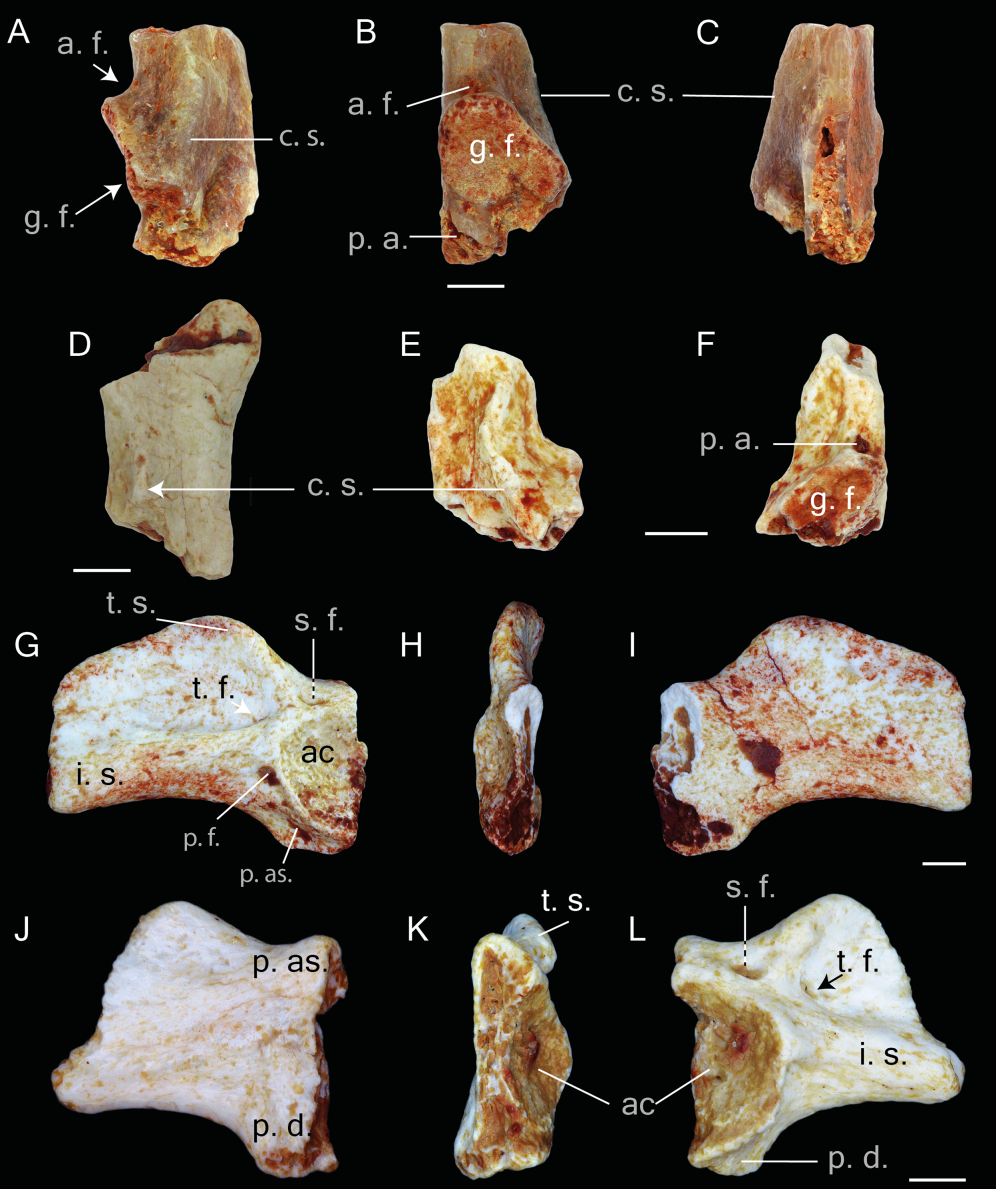
Postcranial elements of *Thaumastosaurus bottii*. (A–C) Left scapula, MJSN VRR006-607, loc. V_p24; (D) left scapula, MJSN VRR006-608, loc. V_p24 and (E–F) right scapula, MJSN VRR006-609, loc. V_p24; (G-I) left ilium, NHMB V.R.195, loc. V_06 and (J-L) right ilium, NHMB V.R.142, loc. V_K72. The bones are figured in (A, D, E) dorsal; (B, F, I, J) medial; (C, G, L) lateral; (H, K) posterior views. Abbreviations: a. f., angular fossa; ac., acetabulum; c. s., crista supraglenoidalis; i. s., iliac shaft; g. f., glenoid fossa; p. a., pars acromalis; p. as., pars ascendens; p. ds., pars descendens; s. f., supraacetabular fossa; p. f., preacetabular fossa; t. f., tubercular fossa; t. s., tuber superior. Scale bars equal 1 mm.

Class **Amphibia** Gray, 1825
Order **Anura** Fischer von Waldheim, 1813
Clade **Ranoides** Frost et al., 2006
Clade **Ranoidea** Frost et al., 2006
Clade **Natatanura** Frost et al., 2006
Genus ***Thaumastosaurus*** De Stefano, 1903
***Thaumastosaurus bottii*** De Stefano, 1903
(Figs. 2–5)

### Material

Premaxilla–LA_86: one right (MGL 101580); V_p24: eight left (VRR006-574, -575, -296, -620); V_p20: one right (MJSN VRR006-570); V_06: two left (NHMB V.R.188–.189) and two right (NHMB V.R.190–.191); V_K72: three right (NHMB V.R. 154–.156) and two left (NHMB V.R.152–.153). Maxilla–LA_86: ten (MGL 101581, 101582, 101610, 101611); LA_83: one left (MGL 101593); V_p24: 22 left (MJSN VRR006-579–-581) and 27 right (MJSN VRR006-582–584), as well as numerous fragments left/right (MJSN VRR006-594); V_p22: one (MJSN VRR006-573); V_p20: one left (MJSN VRR006-413) and left/right (MJSN VRR006-567); V_81: three left/right (NHMB V.R.183–.185); V_06: 13 left (NHMB V.R.259–.271) and 14 right (NHMB V.R.245–.258), 19 left/right bones (NHMV V.R.unnumbered); V_71: one left (NHMB V.R.131) and one right (NHMB V.R.130), two left/right (NHMB V.R.128); V_K72: four left (NHMB V.R.160–.163) and four right (NHMB V.R.164–.167), 17 left /right (NHMV V.R.unnumbered); D_1: one maxilla (PIZUM A/II 120); D_2: one maxilla (PIZUM A/II 121); D_?: one maxilla (PIZUM A/II 125); D_A: one maxilla (PIZUM A/II 126). Nasal–LA_86: two left (MGL 101587); V_71: one left (NHMB V.R.138); V_K72: one left (NHMB V.R.149) and one right (NHMB V.R.148); V_81: one left/right (NHMB V.R.180); V_06: one left (NHMB V.R.244) and one right (NHMB V.R.243); V_p24: one left (MJSN VRR006-597), one right (MJSN VRR006-596), one left/right (MJSN VRR006-598). Frontoparietal–V_K72: two left (NHMB V.R.157–.158) and one right (NHMB V.R.159); V_06: three right (NHMB V.R.239–-241) and 12 left/right (NHMB V.R.242); V_p24: 5 left (MJSN VRR006-577), nine right (MJSN VRR006-578); D_?: one left frontoparietal (PIZUM A/II 120); D_A: one frontoparietal (PIZUM A/II 127). Incomplete skulls–V_St: two incomplete specimens, composed of the prooticooccipitals with co-ossified frontoparietal and parasphenoid (NHMB V.R.31, .37); V_81: one left (NHMB V.R.187). Parasphenoid–V_p24: one (MJSN VRR006-619). Squamosal–LA_86: five left (MGL 101590) and six right (MGL 101588, 101589); V_71: three left (NHMB V.R.135–.137) and two right (NHMB V.R.133, .134); V_K72: three left (NHMB V.R.145–.147), three left/right (NHMB V.R.unnumbered); V_81: two left/right (NHMB V.R.181, .182); V_06: 12 left (NHMB V.R.204–.210; .232–.236) and 23 right (NHMB V.R.211–.231, .237–.238); V_p24: 30 left (MJSN VRR006-585, -587) and 37 right (MJSN VRR006-588–-592); D_?: one left squamosal (PIZUM A/II 122). Squamosal in contact with maxilla–V_p24: one (MJSN VRR006-593). Angular–V_p18: one left/right (MJSN VRR006-566); V_p22: two right (MJSN VRR006-571, -395); V_p24: seven left (MJSN VRR006-610–-612, -618) and eight right (MJSN VRR006-613, -614). Skull bones–LA_86: numerous skull bones (MGL 101591); LA_91: nine skull bones (MGL 101592); V_St: 16 skull bones (NHMB V.R.36); V_K72: 25 skull bones (NHMB V.R.168–.176); V_71: three skull bones (NHMB V.R.139–.141); V_81: three skull bones (NHMB V.R.177–.179); V_p18: numerous skull bones (MJSN VRR006-564); V_p20: six skull bones (MJSN VRR006-568, -569); V_p22: two skull bones (MJSN VRR006-342); V_p24: numerous skull bones (MJSN VRR006-529, -595); V_p29: 13 skull bones (MHSN VRR006-617); D_1: one bone (PIZUM A/II 120); D_A: one skull bone (PIZUM A/II 128). Atlas–LA_86: one (MGL 101583); V_K72: one (NHMB V.R.150); V_06: three (NHMB V.R.196–.198); V_p18: one (MJSN VRR006-563); V_p24: one (MJSN VRR006-576). 2nd–7th vertebrae–V_p24: four vertebrae (MJSN VRR006-530, -601); V_p29: one vertebra (MJSN VRR006-616). 8th vertebra–LA_86: one (MGL 101584); V_K72: one (NHMB V.R.151); V_81: one (NHMB V.R.186); V_06: two (NHMB V.R.199-200); V_p24: four vertebrae (MJSN VRR006-599). Sacral vertebra–LA_86: one (MGL 101585); V_06: one (NHMB V.R.201); V_p24: two (MJSN VRR006-600). Urostyle–LA_86: one (MGL 101586); V_06: two (NHMB V.R.202, .203); V_p24: three (MJSN VRR006-602). Scapula–V_p24: two left (MJSN VRR006-607, -608) and one right (MJSN VRR006-609). Radioulna–V_p18: one (MJSN VRR006-565) and V_p24: four (MJSN VRR006-604). Humerus–V_71: one (NHMB V.R.129). Ilium–LA_86: one left (MGL 101579) and four right (MGL 101578); V_St: one right (NHMB V.R.5); V_71: one left (NHMB V.R.132); V_K72: three left (NHMB V.R.142–.144); V_06: one left (NHMB V.R.192) and three right (NHMB V.R.193–.195); V_p18: one (MJSN VRR006-562); V_p22: one right (MJSN VRR006-572); V_p24: ten left (MJSN VRR006-605)and six right (MJSN VRR006-606); D_?: one right (PIZUM A/II 123).

### Description

The entire material is represented by fragments of different parts of different bones. The material contains elements (at least in the pockets of the La Verrerie de Roches) belonging to individuals of different sizes, which correspond to different ontogenetic stages. The frontoparietals, squamosals, maxillae and nasals show dermal ornamentation on the external surfaces. The ornamentation includes rather shallow and mainly rounded or elongated pits of different sizes. The external surface of the premaxilla is smooth without any ornamentation (Figs. 2A–2D). In the material of the Les Alleveys, some differences in the sculpture of the dermal ossification are observable. The larger individuals resemble the ornamentation described above, whereas those of the small specimens are composed of elongated and ridge-bordered sulci (Figs. 2R–2S).

### Premaxilla.

The paired premaxilla are low and compact. In the ventral view, the bone is slightly curved. In complete specimens, the dental shelf possesses 8–10 tooth pedicles. The medial process is short, and is broad at its base. Ventrally, the triangular medial process possesses a longitudinal crest (Fig. 2B). The presence of the lateral process cannot be proven. Most likely it was present, since the posterior edge of the bone, lateral to the medial process, is prominent. Apparently, in *Thaumastosaurus* from La Verrerie de Roches, these two processes do not have the same size. The hemicylindrical alary process is narrow at its base and broadens distally (Fig. 2B). The distal portion of the process is lacking. On both the lateral and medial sides of the alary process, nutritive foramina are present. They are located in depressions of different size, among which the largest one is the lateral one. The medial edge of the bone, by which it abuts the contralateral premaxilla, is either smooth or possesses some rugosities and a medium–sized foramen.

### Maxilla.

The maxilla is an elongated and low bone with an ornamented dorsal surface. In lateral view, the dorsal edge of the bone between the zygomaticomaxillar and frontal processes, i.e., the margo orbitalis (Figs. 2L–2O), has a concave outline; in larger individuals, it is less concave. A rather weakly pronounced notch is observable in the deepest point of the margo orbitalis (Fig. 2O). The zygomaticomaxillar process has a round outline and projects over the dorsal edge of the ornamented lateral surface (Fig. 2L). In larger bones, corresponding to the older ontogenetic stages, the process is larger, whereas the dorsal edge of the ornamented lateral surface in the area of the zygomaticomaxillar process flattens (Fig. 2N). The margo nasalis of the maxilla is curved (Figs. 2N and 2Q), and its lower portion is broad and nearly fused with the horizontal lamina. In labial view, a well-pronounced horizontal lamina is visible. Anteriorly, it projects anterodorsally and builds an anterior spine (Fig. 2Q). In lateral view, it projects over the anterior edge of the margo nasalis. Behind the anterior spine, the medial face of the lamina is convex, but it is flat in the area of the frontal process. Seen in dorsal view, at the level of the margo orbitalis, the horizontal lamina is broad and reaches its greatest width (Fig. 2O). Thereby, a surface forms, which is either flat (smaller individuals) or possesses a groove for the palatoquadrate bar (larger individuals). Anteriorly, this groove turns into another shallow groove between the frontal process and recessus vaginiformes. Only the lower portions of these two elements are preserved.

At the anterior base of the frontal process, two small nutritive foramina are present. The horizontal lamina, below the zygomaticomaxillar process, projects into a prominent pterygoid process (Figs. 2M and 2O). The process abuts posteriorly on a round, well-pronounced, posteriorly facing pit. Behind the pit, the horizontal lamina narrows significantly and continues as a gradually diminishing strip, which bends ventrally to the ventral edge of the maxilla and borders the posterior margin of the dental shelf. The dental shelf on the maxilla runs nearly parallel to the outline of the horizontal lamina, possessing 41 tooth pedicles in the most complete specimen. Anteriorly, the dental shelf starts before the beginning of the horizontal lamina. The most anterior triangular flat surface of the bone (articulation facet for the premaxilla (Roček & Lamaud, 1995) separates them (Fig. 2Q).

### Nasal.

The nasal bones are thin and fragile. The bone lateral surface bears a dermal ornamentation. Overall, eight fragments from different parts of the bones are preserved. The margo orbitalis has a concave outline. In posterior view, it is thicker than its anterior portion and possesses a shallow posterior groove running parallel and below the posterior margin of the dorsal sculptured surface of the bone (Figs. 2I–2J). The lateral part of the bone is partially preserved. Laterally, it reduces and transforms into a narrow paraorbital process (Figs. 2E–2H). The paraorbital process bears a longitudinal elongate surface lacking the dermal ornamentation. This surface serves as a ventral wall for the groove for the ductus nasolacrimalis and for the attachment of the anterior tip of the squamosal bone (Fig. 2E). The anterior margin of the paraorbital process (margo maxillaris) articulates with the frontal process of the maxilla. In ventral view of the bone, the articulation facet with maxilla is well visible. Medially, the anterior margin of the nasal frames the posterior border of the margo nasalis. In the available fossil material, only the lateral portion of the margo nasalis is preserved. It is slightly curved and possesses a prominent anteriorly directed parachoanal process, best observable in the ventral view (Figs. 2G–2H). In ventral view, a rather deep cavity at the posteromedial part of the nasal is observable. It is posteriorly delimited by the flange of the margo orbitalis and medially by the margo medialis.

### Frontoparietal.

The description of the frontoparietals is based on isolated fragments (Figs. 2T–2Y), as well as those in association with prooticooccipital bones (NHMB V.R.31, V.R.37, Figs. 3A–3B). All of the elements possess an ornamented dorsal surface. The clear boundaries (sutures) of the frontoparietal and prooticooccipital cannot be followed; because of this, the reconstruction of the bone elements of the mummy frog (Laloy et al., 2013) is used as reference. Although the frontoparietals are represented by isolated left and right parts, the partially complete skulls show both sides of the bone to be fused into each other. The margo orbitalis of the frontoparietal extends laterally and forms the tectum supraorbitalis (Figs. 2V and 2W). Anteriorly, the tectum supraorbitalis is elongate and narrow, posteriorly it is shorter and thicker. In ventral view, a medially concave groove runs at the tectum supraorbitalis. The base of the anterior half of the groove runs parallel to the pars contacta (Figs. 2U and 2W). The pars contacta is incompletely preserved, it is straight in its outline and only curves laterally in its posterior portion. Medially from the pars contacta, the ventral face of the frontoparietal possesses an elongated oval, paired larger facies cerebralis anterior (Figs. 2U and 2W) and a circular, also paired, but smaller, facies cerebralis posterior (Fig. 2Y). The most anterior area of the ventral surface of the bone is covered by longitudinal narrow grooves, corresponding to the articulation surface with the sphenethmoid. In lateral view, the lateral surface of the margo orbitalis is oblique, and it is more sharply bent posteriorly from the tectum supracerebralis. The margo prootica, in the posterior portion of the bone, can be either concave or straight, likely corresponding to the different ontogenetic stage (Figs. 2V–2Y).

### Prooticooccipital.

The paired prooticooccipitals are compact and robust. They are fused together and dorsally covered by the frontoparietal and ventrally supported by the parasphenoid (Figs. 3A and 3B). The foramen magnum is large and dorsoventrally slightly compressed, which makes it oval in outline. Two separate drop-shaped, laterodorsally extending occipital condyles are observable on the ventral margin of the foramen magnum (Fig. 3C). Laterally, the condyles are connected to the prominentia ducti semicircularis posterior by a short slightly prominent horizontal ridge. This ridge and lateral margin of the occipital condyle overlap the foramen jugulare, located in a deep pit. Dorsally to the short horizontal ridge, a rather deep depression is present, which is delimited by the ventromedially projecting, slightly curved median keel. It starts dorsally from the central part of the p.d.s.p. and meets ventrally with the foramen magnum (Figs. 3C and 3I). The keel is well-pronounced in larger individuals–weakly developed in NHMB V.R.31 (Fig. 3C) and clearly visible in NHMB V.R.37 (Fig. 3I). In dorsal view, the medial portion of the prooticooccipital is massive and wide, the dorsal surface is concave and decreases posteriorly. The otic capsule is narrower and possesses a prominent parotic crista (Fig. 3A). The lateral parts of the bone are damaged and expose the fenestra ovalis. In anterior view, the prooticooccipital is separated by a laterodorsally projecting groove for the vena jugularis interna into massive high ventral and more slender dorsal parts (Fig. 3I). The medial end of the groove is pierced by the foramen prooticum. Whether the latter foramen is subdivided cannot be observed in the available material. The posterior margin of the bone possesses two longer lateral paraoccipital processes and one medial shorter posterior superior process (Fig. 3C). The bone margin at both the left and right sides from the posterior superior process are concave. Below, at the deepest point of the curve, several distinct pits are observable. Below both posteromedial corners of the paraoccipital processes, small foraminae arteriae occipitalis are located. Posteriorly, the paraoccipital processes are located close to the posterolaterally directed prominentia ducti sedmicircularis posterior (p.d.s.p.) of the prooticooccipital bone (Figs. 3A and 3C). On both sides, they project over the posterior margins of the prooticooccipital bones.

### Squamosal.

The preserved parts of the bones show the following morphology: in lateral view, the bone is arch–shaped; its distal part, posteriorly from the ramus paroticus/posterolateral process, is oval or rounded in shape; at the position of the posterolateral process, the bone reaches its most narrow portion; and anteriorly, it broadens again. In medial view, the posterior portion of the squamosal possesses two flanges running subparallel to the bone outline. Anteriorly, they fuse and transform into a posterolateral process (Figs. 4B and 4D). Between these flanges, the bone surface is concave and slightly sculptured, and it builds the lateral wall of the tympanic cavity. The upper flange—ramus paroticus projects horizontally (Figs. 4A–4B). The ramus paroticus is low at the most posterior portion and sharply extends medially in the area of the posterolateral process. The anterior portion of the squamosal is elongate (Figs. 4G–4J), the dorsal surface of this bone portion is broad, flat or concave, forming the bottom of the orbit—margo orbitalis. In lateral view, the margo orbitalis is concave, and a maxillary process is visible at the anterior portion of the bone, which projects ventrally and is higher in its posterior portion. The ventral edge of the bone is concave behind the maxillary process. The anteroventral part of the maxillary process possesses a round articulation surface with the zygomaticomaxillar process of the maxillae. In medial view, a horizontal lamina (Fig. 4H) of different degrees of development is present ventrally from the margo orbitalis. The maxillary process terminates posteriorly by a shallow incisura. Above it, in the middle part of the bone, a circular facet for articulation with (?) the pterygoid is present (Fig. 4H). Behind it, the bone increases gradually in its height. In medial view, a shallow depression of the bony surface is observable.

### Angular.

The bone is slender and curved. The Meckelian groove passes laterodorsally along the entire bone length. Behind the coronoid process, it runs dorsally and reaches its maximal width. The coronoid process is semilunar, and its lateral edge projects slightly over the Meckelian groove (Fig. 4M). In the area of the coronoid process, the lateral surface of the bone possesses a short horizontal groove (Fig. 4K).

### Vertebral column.

The atlas is fragmentary, and the neural arch is lacking in all specimens. Anteriorly, two elongated cervical cotyles are present. They are not fused to each other but are connected with a narrow flange. The dorsal portion of the cotyle is straight and the ventral one is curved. The preserved vertebrae can be classified by the centrum morphology to the procoelous, amphicoelous and biconvex. The procoelous vertebrae can be identified as the II–VII vertebrae, the amphicoelous as the VIII vertebra and the biconvex, with two posterior and one anterior condyles, as the sacral vertebrae. This arrangement of the vertebrae morphology is characteristic for the diplasiocoelus configuration of the vertebral column. In ventral view, both lateral sides of the vertebrae centra can bear a short longitudinal groove, and no foramina can be observed. The apophyses and neural arch are not preserved. The urostyle fragments show two distinct anterior condyles that do not merge together. Above them, the neural crest was present, which is preserved only by its thin ventral walls.

### Scapula.

The bone is present either by the proximal (two specimens) (Figs. 5A–5D) and middle (Figs. 5E–5F) portions (corpus scapula) (one specimen). The proximal portion possesses a large oval glenoid fossa, projecting posterolaterally over the corpus scapula (Fig. 5A). The glenoid fossa is separated from the pars acromialis by a notch (Fig. 5A). In the area of the glenoid fossa, the external surface of the bone is slightly concave. In dorsal view, a well-pronounced, curved crista supraglenoidalis can be observed. The bone surface between the crista supraglenoidalis and the ridge of the glenoid fossa is concave. Anteriorly, the crista descends sharply and is confluent with the pars acromialis (Fig. 5A). The latter is flat and preserved only by its base. This preservation allows the presumption about a well-pronounced and anteriorly extended distal portion of the pars acromialis. The base of the lateral wall of the glenoid fossa bears a small angular fossa (Fig. 5B). The corpus scapula narrows in its middle part, where the lateral extension of the crista supraglenoidalis also terminates (Figs. 5E–5F). It is situated at the posterior half the bone.

### Ilium.

This pelvic bone is preserved. Both the partes ascendens and descendens are damaged. The base of the former is narrower than the later. The partly preserved acetabulum has concave surface. It is lacking its posterior part (Figs. 5G and 5L). The acetabular margin reaches the maximal height at its anteroventral edge (Figs. 5K and 5H). The tuber superior is prominent and elongate. It is broad at the base and gradually narrows dorsally. Anteriorly, it abuts an anteriorly descending dorsal crest. Both basal corners of the tuber superior are pierced by fossa. The posterior one-supraacetabular fossa is larger than the anterior one. The anterior one-tubercular fossa is located in a shallow depression (corner) between the iliac shaft and tuber superior. At the base of the iliac shaft, above the preacetabular zone and directly below the tubercular fossa, the preacetabular fossa is observable (Figs. 5G and 5L). The medial surface of the corpus ossi between the partes ascendens and descendens has a triangular form and bends (deepens) posteriorly (Figs. 5I and 5J).

### Comparison and remarks

The entire studied material from four localities referable to frogs, with the exception of three ilia referable to other frog families (see below), can be assigned to the genus *Thaumastosaurus* De Stefano 1903 (Rage & Roček, 2007) by: (1) a hyperossified skull with an ornamented dermal bones (frontoparietal, nasal, maxilla, and squamosal); (2) both paired nasals and frontoparietals co-ossified, as well as nasal with the sphenethmoid and frontoparietals with the prooticooccipital correspondingly; (3) the lack of a contact between the squamosal and frontoparietal; (4) merged posteriolateral process and ramus paroticus of squamosal, articulating with the crista parotica of the otic capsule; and (5) the characteristic morphology of the posterior surface of the skull, including the presence and position of the foramina.

Although the bone material comes from different samples and localities (likely representing different stratigraphic ages), no morphological differences can be observed. The comparison with the material of the four known species of *Thaumastosaurus* revealed strong similarities with the species *T. bottii* by having: (1) a comparable dermal ornamentation with shallower pits, relatively thicker and varying in width ridges lacking the tubercles; (2) a notch on the margo orbitalis of the maxilla, however, the notch is less pronounced than in the previously known material of *T. bottii* (Rage & Roček, 2007; Laloy et al., 2013); and (3) the presence of the well-developed groove for the vena jugularis interna in the anterior wall of the prootic.

*T. bottii* is considered to differ from *T. gezei* by having a shorter anterior extension of the squamosal, which separates the maxilla from the orbit. To state this in the Swiss *Thaumastosaurus*, an articulated and better-preserved material is necessary. The premaxilla of *Thaumastosaurus* from Verrerie de Roches, Les Alleveys and Dielsdorf can be distinguished from that of the Quercy mummy of *T. gezei* by: (1) a short and robust medial process; (2) medial and lateral processes unequal in size; and (3) the presence of the ventral longitudinal ridge on the medial process (Fig. 3B) (no ventral longitudinal ridge can be observed in *T. gezei* from Quercy). The premaxilla is missing in all the other three species of the genus, making the comparison of this bone impossible.

The species *T. sulcatus* differs from the Swiss *Thaumastosaurus* by having, e.g., a dermal ossification composed of elongate and ridge-bordered sulci and a thinner and flatter lamina horizontalis (Holman & Harrison, 2002). The first character should be regarded tentatively, since on one hand, the material available for the erection of the species is represented by few specimens, and on the other hand, the same morphology is found in the *Thaumastosaurus* material from the Les Alleveys locality, which is presented by a small-sized/subadult individual.

The studied material can be distinguished from *T. wardi* (Holman & Harrison, 2003) by: (1) a rounded lamina horizontalis of the maxilla; (2) shallower pits of the dermal ornamentation on the squamosal; and (3) an unevenly curved and not hemicircular orbital margin of the squamosal.

Another distinguishing characteristic for species has been suggested (Laloy et al., 2013): the form of the base of the processus paroticus (ramus paroticus) of the squamosal, which is almost straight in *T. gezei* and curved in *T. wardi*. This characteristic should be revised in the original material, since in *T. wardi,* this character cannot be clearly observed due to the incomplete preservation of the processes. In the studied material, the base of the processus is straight (Figs. 4C2 and 4D2).

In addition to the cranial bone, the studied *Thaumastosaurus* material from Swiss localities is also provided with postcranial elements of the skeleton. The diplasiocoelous configuration of the vertebral column (procoelous II–VII vertebrae, amphicoelous VIII vertebra, biconvex sacral vertebra, and urostyle with two anterior condyles) suggests its attribution to the clade Ranoidea. Although the scapula is not completely preserved, it strongly resembles that of the mummy MNHM.F.QU17279 (Laloy et al., 2013) and *Thaumastosaurus* sp. UM–ECA2537 (Rage, 2016) .

The most interesting is the finding of 31 iliac specimens from three studied localities that display features characteristic for the clade of Ranoidea: the presence of the tubercular fossa; high tuber superior; and an anteriorly decreasing in height dorsal crest (Sanchíz, 1998; Gómez & Turazzini, 2015). Ilia resembling the same morphology of Eocene ranids/ranoids are found in numerous localities of MP16–MP19 ages (Table S1). Earlier, only three ilia have been referred in the literature to the genus *Thaumastosaurus* (*Thaumastosaurus wardi*, Hordle Cliff near Milford on Sea; SW Headon Hill (Headon Hill 3) and Rodent Bed localities, England (Holman & Harrison, 2003)), showing only the base of the diminished dorsal prominence, as well as no dorsal crest. It is remarkable that no trace of a diminished dorsal crest on the iliac shaft can be observed on the illustrated bone (Holman & Harrison, 2003). The published three ilia from Headon Hill 3 most likely belong to another frog taxon (not to Ranoidea) and cannot be referred to the genus *Thaumastosaurus*. The only ilium belonging unquestionably to *Thaumastosaurus* can be considered that of the mummy specimen (Laloy et al., 2013), which is only preserved by its anterior part, making it impossible to compare with the Swiss finds. It is important to note, that aside from 31 ilia of ranoid morphology, the studied Swiss localities (samples V_06 and V_K72) provided three further ilia showing the morphology of the Bufonidae (two ilia) and Pelobatidae (one ilium) families. Moreover, no further bones from the studied material can be referred to these families. Taking these arguments into consideration, the assignment of the ilia, resembling Ranoidea morphology found in Swiss localities, to the genus *Thaumastosaurus* seems most likely.

The anuran fauna of Europe from the late middle–late Eocene (MP16–MP20) was dominated by ranoid frogs: the genus *Thaumastosaurus* and two (Rage, 1984) or three ranoid frogs (Rage, 2012; Rage, 2016) (*Rana* sp. (Rage, 1984), as cf. *Rana* sp. (Rage, 2012), Ranidae indet. (Roček, 2013)). Two/three ranoid taxa are distinguished by sized and some morphological differences (Rage, 1984; Rage, 2016), which could represent different ontogenetic stages and be sexual dimorphism and not necessarily different species. The rest of the Ranoidea are represented by postcranial elements, including vertebrae, humeri, ilia, and coracoids (Rage, 1984). Recent studies (Laloy et al., 2013; Rage, 2016) showed that some elements, excluding ilia and humeri, can, in fact, be assigned to *Thaumastosaurus*. Since the ilium was not completely preserved in the recently described mummy from Quercy, the earlier reported isolated ilia (Rage, 1984; Rage, 2016), also cannot be assigned to it (Rage, 2016). However, the new finds from La Verrerie de Roches, Les Alleveys and Dielsdorf suggest that ilia of the Ranoidea indet. (cf. *Rana* sp.) (resembling the morphology figured in (Rage, 1984) should be referred to *Thaumastosaurus*. Apart from this, the following further facts also argue for this assignment: these two frogs have the same stratigraphic record–they appeared in the basal MP16, the first half of Bartonian in Switzerland (localities of Les Alleveys, and La Verrerie de Roches); and “co-occur” in nearly all localities until MP19 (MP20?), terminal Priabonian. It would be remarkable if, in the same locality or even in the same time period, one taxon (*Thaumastosaurus*) would be represented by the elements of the skull, and the second one (Ranoidea indet.) by elements of the posterior part of the postcranial skeleton.

Taking into account the morphological differences, as well as similarities, both cranial and postcranial frog material from the localities of La Verrerie de Roches, Les Alleveys and Dielsdorf can be assigned to the species *Thaumastosaurus bottii* with the exception of the ilia belonging to Bufonidae and Pelobatidae.

## Discussion

The present paper presents the most eastern occurrence of the genus *Thaumastosaurus*, found in three localities in Switzerland. The material from the studied localities referred as *Thaumastosaurus bottii* so far represent the oldest record of this species, as well as the first appearance of the genus in Western Europe (Table S1). Among them, Verrerie de Roches provides the most abundant material, and the least abundant material is found in the locality Dielsdorf. All the material herein studied is represented by fragments of different bones. The preserved bone material (at least in the pockets of the La Verrerie de Roches) contains elements belonging to individuals of different sizes, which can be referred to different ontogenetic stages. This suggests the presence of the autochthonous natural resident population of *Thaumastosaurus bottii* in the La Verrerie de Roches pocket nr. 24.

**Figure 6:**
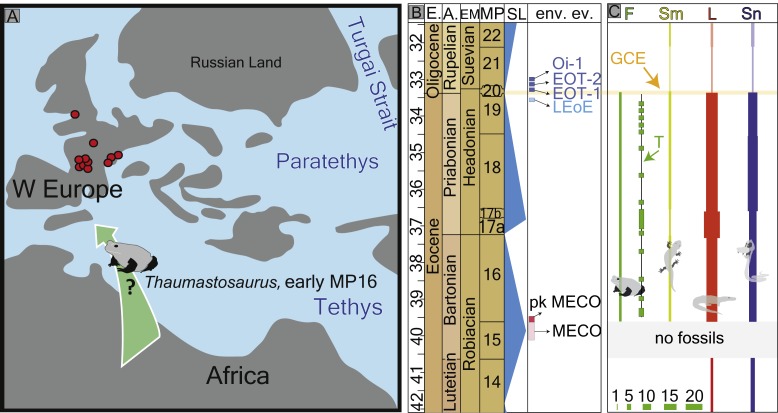
Palaeogeography of Europe with fossil record of the late Eocene–early Oligocene record of herpetofauna (A) palaeogeographic map Europe of Eocene redrawn by Popov et al. (2004) with the location of the fossil localities with *Thaumastosaurus* (in red), indicating most probable migration route. (B) see Fig. 1B. (C) diversity changes (number of species) of frog, salamander, lizard and snake fossil records in the Quercy area (Rage, 2012) with indication (horizontal yellow balk) of the Grande Coupure Event; the thickness of the lower left green bulks corresponds to the numerical values of species numbers indicated there. The heading abbreviation in the section C as follows: F, frogs; Sm, salamanders; L, lizards; Sn, snakes; T, *Thaumastosaurus* fossil record; GCE, Grand Coupure Event.

The first appearance of the Western European endemic genus *Thaumastosaurus* coincides with the base of the MP16, at least considering the localities Les Alleveys and La Verrerie de Roches (the karstic pockets of Dielsdorf have less precise ages). This time period is characterised by ∼4–6 °C worldwide total warming of both surface and deep oceanic waters (Bohaty et al., 2009; Boscolo Galazzo et al., 2014). This event is known as the Mid-Eocene climatic optimum (MECO) and lasted only 500 ky. After the short warming event, the climate continued to cool gradually in stepwise manner, which resulted in the Eo–Oligocene transition (EOT) by an onset of the building of a permanent Antarctic ice-sheet (Katz et al., 2008; Vandenberghe, Hilgen & Speijer, 2012; Boscolo Galazzo et al., 2014) (Figs. 1 and 6). The latest secure appearance of *Thaumastosaurus bottii* is recorded from the Escamps locality (MP19) in France (Rage, 2006) (Table S1) shortly before the late Eocene cooling event (the genus is perhaps also present in a locality in MP20). The first half of the *Thaumastosaurus* occurrence time interval (34–40 Ma) is characterised by tropical evergreen forests under warm and humid conditions, whereas the second half is characterised by an environment with a relatively drier climate, probable appearance of a dry season, and more open vegetation corresponding to woodland savannah. From the EOT onwards, the picture changed from (sub)desert to light forest environments, typically with more arid conditions and relatively low mean annual temperature (Escarguel, Legendre & Sigé, 2008). The global cooling and, following that, major changes in the environment resulted in the large–scale extinction of many endemic vertebrate groups in Europe on one hand, and on another hand was followed by the early Oligocene palaeogeographic reorganisation in Eurasia and connection of different separated landmasses of Europe and Asia (Legendre, 1989; Meng & McKenna, 1998; Rage, 2012; Vandenberghe, Hilgen & Speijer, 2012). These dramatic faunistic changes at the Eo–Oligocene transition are known, at least in Europe, as the Grande Coupure event (Stehlin, 1909) (Fig. 6C). In the larger geographical scale, the synchronicity of this event has been questioned in different publications (Meng & McKenna, 1998; Vandenberghe, Hilgen & Speijer, 2012).

Since not much is known about the herpetofauna of MP15 (before the stratigraphically oldest *Thaumastosaurus* record), it is impossible to confidently exclude the genus arrival in MP15. Due to this, as well as the lack of a fossil record of the genus outside of Europe, it is difficult to conclude about where (and when exactly) the genus *Thaumastosaurus* arrived at the European continent from. Nevertheless, its clear relationship with the pyxicephalid ranoid frogs, having an African origin, has been suggested (Laloy et al., 2013; Rage, 2016).

Little is known about the intercontinental faunistic exchanges before or at the beginning of the MP16. Only one primate group (Amphipithecidae) suggests the presence of a possible connection between Africa and Europe at the Lutetian/Bartonian boundary (Gheerbrant & Rage, 2006). However, the presence of a small faunistic turnover in herpetofaunistic assemblages has already been suggested in previous studies (Delfino, Rage & Rook, 2003; Rage, 2012). The faunistic dispersal from Africa into Western Europe is, so far, the most sensible scenario to explain their distribution, although during MP15 and MP16, the global sea level rose (Fig. 6B), making the existence of land bridges connecting Europe and Africa less plausible. Another possible dispersal route can be considered from the Indian subcontinent via Western Asia and Eastern European land masses into Western Europe. However, this scenario is hypothetical, since the genus does not have any record across this area (Folie et al., 2013; Rage, 2016).

## Conclusions

Taking into consideration the first and last appearances of *Thaumastosaurus,* as well as palaeogeographic situation and climatic events before, during and after the period when *Thaumastosaurus* was present in Europe, the following can be concluded:

the genus *Thaumastosaurus* entered Western Europe from Africa during/after the peak of the Mid-Eocene climatic optimum, when humid and warm conditions prevailed;

later, due to the gradual cooling of the global climate, the climate became drier and colder, and the genus *Thaumastosaurus* became extinct and did not survive the Grande Coupure event.

The present study allows the conclusion that the true frogs were absent in the Eocene of Europe, suggesting their later arrival in the earliest Oligocene (*Pelophylax* sp., locality Möhren 13, Germany (Sanchíz, Schleich & Esteban, 1993) when Europe was colonised by clades arriving from Asia. In addition to the water frogs, further amphibians (e.g., *Chioglossa* sp. from Möhren 6 and cf. *Tylototriton* sp. from Möhren 13, Germany, *Latonia* sp. from Möhren 12 and Gräffmühle 10 (Böhme, 2010) invaded Europe in the earliest Oligocene after the Grande Coupure extinction event. The present hypothesis about the dispersal/appearance of Ranidae could be considered by molecular genetic studies for recalibration of the molecular clock.

## Institutional/Collection Abbreviations

MGL: Musée cantonal de géologie, Lausanne, Switzerland
MJSN: JURASSICA Museum (former Musée jurassien des sciences naturelles, MJSN), Porrentruy, Switzerland
NHMB: Naturhistorisches Museum Basel, Basel, Switzerland
PIMUZ: Paläontologisches Institut und Museum der Universität Zürich, Zurich, Switzerland

##  Supplemental Information

10.7717/peerj.5511/supp-1 Table S1 Table summarizing the localities with fossil record of Thaumastosaurus and Ranidae / Rana sp. / ? Rana sp Abbreviations: cor, coracoid; hum, humerus; il, ilium; svert, sacral vertebra; psvert, praesacral vertebra, T, Thaumastosaurus, R, Ranidae / Rana sp. / ? Rana sp. The localities (22–24) studied in the present paper are in bold.

## Additional Information and Declarations

### Data Availability

The following information was supplied regarding data availability: The specimens/material described in the manuscript are stored in: MGL, Musée cantonal de géologie, Lausanne, Switzerland; MJSN, JURASSICA Museum (former Musée jurassien des sciences naturelles, MJSN), Porrentruy, Switzerland; NHMB, Naturhistorisches Museum Basel, Basel, Switzerland; and PIMUZ, Paläontologisches Institut und Museum der Universität Zürich, Zurich, Switzerland. The list is indicated in the section “material and methods” and “described material” of the manuscript.

## References

[ref-1] Becker D, Rauber G, Scherler L (2013). New small mammal fauna of late Middle Eocene age from a fissure filling at La Verrerie de Roches (Jura, NW Switzerland). Revue de Paléobiologie.

[ref-2] Bohaty SM, Zachos JC, Florindo F, Delaney ML (2009). Coupled greenhouse warming and deep-sea acidification in the middle Eocene. Paleoceanography.

[ref-3] Böhme M (2003). The miocene climatic optimum: evidence from ectothermic vertebrates of Central Europe. Palaeogeography, Palaeoclimatology, Palaeoecology.

[ref-4] Böhme M (2010). Ectothermic vertebrates (Actinopterygii, Allocaudata, Urodela, Anura, Crocodylia, Squamata) from the Miocene of Sandelzhausen (Germany, Bavaria) and their implications for environment reconstruction and palaeoclimate. Paläontologische Zeitschrift.

[ref-5] Boscolo Galazzo F, Thomas E, Pagani M, Warren C, Luciani V, Giusberti L (2014). The middle Eocene climatic optimum (MECO): a multiproxy record of paleoceanographic changes in the southeast Atlantic (ODP Site 1263, Walvis Ridge). Paleoceanography.

[ref-6] Coxall HK, Wilson PA (2011). Early Oligoceme glaciation and productivitiy on the eastern equatorial Pacific: insights into global carbon cycling. Paleoceanography.

[ref-7] De Stefano G (1903). I Sauri del Quercy apparenenti alla collezione Rossignol. Atti della Socieita Italiana di Scienze Naturali.

[ref-8] Delfino M, Rage J-C, Rook L, Reumer JWF, Wessels W (2003). Tertiary mammal turnover phenomena: what happened to the herpetofauna. Distribution and migration of Tertiary mammals in Eurasia.

[ref-9] Escarguel G, Legendre S, Sigé B (2008). Unearthing deep-time biodiversity changes: the Palaeogene mammalian metacommunity of the Quercy and Limagne area (Massif Central, France). Comptes Rendus Geoscience.

[ref-10] Fischer von Waldheim G (1813). Zoognosia. Tabulis Synopticis Illustrata, in Usum Prælectionum AcademiæImperialis Medico-Chirurgicæ Mosquensis Edita.

[ref-11] Folie A, Rana RS, Rose KD, Sahni A, Kumar K, Singh L, Smith T (2013). Early Eocene frogs from Vastan Lignite Mine, Gujarat, India. Acta Palaeontologica Polonica.

[ref-12] Frost DR, Grant T, Faivovich J, Bain RH, Hass A, Haddad CFB, De Sá RO, Channing A, Wilkinson M, Donnellan SC, Raxworthy CJ, Campbell JA, Blotto BL, Moler PE, Drewes RC, Nussbaum RA, Lynch JD, Green D, Wheeler WC (2006). The amphibian tree of life. Bulletin of the American Museum of Natural History.

[ref-13] Gheerbrant E, Rage J-C (2006). Paleobiogeography of Africa: how distinct from Gondwana and Laurasia?. Palaeogeography, Palaeoclimatology, Palaeoecology.

[ref-14] Gómez RO, Turazzini GF (2015). An overview of the ilium of anurans (Lissamphibia, Salientia), with a critical appraisal of the terminology and primary homology of main ilial features. Journal of Vertebrate Paleontology.

[ref-15] Gray JE (1825). A synopsis of the genera of reptiles and Amphibia, with a description of some new species. Annals of Philosophy.

[ref-16] Holman AJ, Harrison DL (1999). *Rana (Amphibia: Ranidae)* from the upper Eocene (MP17a) Hordle cliff locality, Hampshire, England. Palaeovertebrata.

[ref-17] Holman AJ, Harrison DL (2002). A New *Thaumastosaurus* (Anura: Familia Incertae Sedis) from the Late Eocene of England, with Remarks on the Taxonomic and Zoogeographic Relationships of the Genus. Journal of Herpetology.

[ref-18] Holman AJ, Harrison DL (2003). A new helmeted frog of the genus *Thaumastosaurus* from the Eocene of England. Acta Palaeontologica Polonica.

[ref-19] Hooker JH, Weidmann M (2000). The Eocene mammal faunas of Mormont, Switzerland. Schweizerische Paläontologische Abhandlungen.

[ref-20] Hooker JJ, Weidmann M (2007). A diverse rodent fauna from the middle Bartonian (Eocene) of Les Alleveys, Switzerland: snapshot of the early theridomyid radiation. Swiss Journal of Geosciences.

[ref-21] Katz ME, Miller KG, Wright JD, Wade BS, Browning JV, Cramer BS, Rosenthal Y (2008). Stepwise transition from the Eocene greenhouse to the Oligocene icehouse. Nature Geosciences.

[ref-22] Laloy F, Rage J-C, Evans SE, Boistel R, Lenoir N, Laurin M (2013). A re-interpretation of the Eocene Anuran *Thaumastosaurus* based on MicroCT examination of a ‘Mummified’ specimen. PLOS ONE.

[ref-23] Legendre S (1989). Les communantés de mammiféres du Paléogène (Eocène supéreur et Oligocène) d’Europe Occidentale: structures, milieux et évolution. Münchner Geowissenschaftliche Abhandlungen (A).

[ref-24] Meng J, McKenna MC (1998). Faunal turnovers of Paleogene mammals from the Mongolian Plateau. Nature.

[ref-25] Minwer-Barakat R, Marigó J, Becker D, Costeur L (2017). A new primate assemblage from La Verrerie de Roches (Middle Eocene, Switzerland). Journal of Human Evolution.

[ref-26] Popov SV, Rögl F, Rozanov AY, Steininger FF, Shcherba IG, Kovac M (2004). Lithological-Paleogeographic maps of Paratethys. Courier Forschungsinstitut Senckenberg.

[ref-27] Rage J-C (1984). Are the Ranidae (Anura, Amphibia) known prior to the Oligocene?. Amphibia-Reptilia.

[ref-28] Rage J-C (2006). The lower vertebrates from the Eocene and Oligocene of the phosphorites du Quercy (France): an overview. Strata.

[ref-29] Rage J-C (2012). Amphibians and squamates in the Eocene of Europe: What do they tell us?. Palaeobiodiversity and Palaeoenvironments.

[ref-30] Rage J-C (2016). Frogs (Amphibia, Anura) from the Eocene and Oligocene of the phosphorites du Quercy (France). An overview. Fossil Imprint.

[ref-31] Rage J-C, Augé ML (2003). Amphibians and squamate reptiles from the lower Eocene of Silveirinha (Portugal). Ciências da Terra (UNL).

[ref-32] Rage J-C, Ford RLE (1980). Amphibians and Squamates from the Upper Eocene of the Isle of Wight. Tertiary Research.

[ref-33] Rage J-C, Roček Z (2007). A new species of *Thaumastosaurus* (Amphibia: Anura) from the Eocene of Europe. Journal of Vertebrate Paleontology.

[ref-34] Roček Z (2013). Mesozoic and Tertiary Anura of Laurasia. Palaeobiodiversity and Palaeoenvironments.

[ref-35] Roček Z, Lamaud P (1995). *Thaumastosaurus bottii* De Stefano, 1903, an anuran with Gondwanan affinities from the Eocene of Europe. Journal of Vertebrate Paleontology.

[ref-36] Rosselet C (1991). Die Fauna der Spaltenfüllunge von Dielsdorf (Eozän, Kanton Zürich). Documenta Naturae.

[ref-37] Rosselet C (1993). The fauna from the fissure fillings of Dielsdorf (Eocene, Canton Zurich, Switzerland). Kaupia Darmstädter Beiträge zur Naturgeschichte.

[ref-38] Sanchíz B (1998). Salientia.

[ref-39] Sanchíz B, Schleich H-H, Esteban M (1993). Water frogs (Ranidae) from the Oligocene of Germany. Journal of Herpetology.

[ref-40] Stehlin HG (1909). Remarques sur les faunules de Mammifères des couches éocènes et oligocènes du Bassin de Paris. Bulletin de la Société Géologique de France.

[ref-41] Vandenberghe N, Hilgen FJ, Speijer RP, Gradstein FM, Ogg JG, Schmitz MD, Ogg GM (2012). The paleogene period. The geologic time scale 2012.

[ref-42] Winter M, Fiedler W, Hochachka WM, Koehncke A, Meiri S, de La Riva I (2016). Patterns and biases in climate change research on amphibians and reptiles: a systematic review. Royal Society Open Science.

[ref-43] Zachos JC, Dickens GR, Zeebe RE (2008). An early Cenozoic perspective on greenhouse warming and carbon-cycle dynamics. Nature.

