# Supplementary material for: Eocene Western European endemic genus Thaumastosaurus: new insights into the question “Are the Ranidae known prior to the Oligocene?”
Source: PeerJ. 2018 Aug 27;6:e5511. doi: 10.7717/peerj.5511 (PMC6118198; doi:10.7717/peerj.5511)
Supplement: Table S1 — Abbreviations: cor, coracoid; hum, humerus; il, ilium; svert, sacral vertebra; psvert, praesacral vertebra, T, Thaumastosaurus, R, Ranidae / Rana sp. / ? Rana sp. The localities (22–24) studied in the present paper are in bold. [file peerj-06-5511-s001.docx]

Table S1. Table summarizing the localities with fossil record of *Thaumastosaurus* and Ranidae / *Rana* sp. / ? *Rana* sp. Abbreviations: cor, coracoid; hum, humerus; il, ilium; svert, sacral vertebra; psvert, praesacral vertebra, T, Thaumastosaurus, R, Ranidae / *Rana* sp. / ? *Rana* sp. The localities (22-24) studied in the present paper are in bold.

| nr. | |  | |  | |  | | |  | | T | | | R | | | *Thaumatosaurus* | | | | | | | | | | | | | | | Ranidae / *Rana* sp. / ? *Rana* sp. | | | | | | | | | |  |
| --- | --- | --- | --- | --- | --- | --- | --- | --- | --- | --- | --- | --- | --- | --- | --- | --- | --- | --- | --- | --- | --- | --- | --- | --- | --- | --- | --- | --- | --- | --- | --- | --- | --- | --- | --- | --- | --- | --- | --- | --- | --- | --- |
|  | | | Locality | | country | | age | reference | | | | |  | | |  | | | species | | | element | | | il | | | figured | | | reference | |  | | element | | il | | figured | | reference | |
| 1 | | Quercy Phosphorite | | France | | MP15-MP19(MP20?) | | | ^1^ | |  | | |  | | | *gezei*, sp. | | | skull | | | - | | | Figs 1, 2 | | | ^1^ | | | - | | - | | - | | - | | ^2^ | |  |
| 2 | | Escamps | | France | | MP19 | | | ^3^ | |  | | |  | | | *bottii* | | | - | | | - | | | - | | | ^4,5^ | | | + | | - | | - | | - | | ^5,6^ | |  |
| 3 | | Rosières 1 | | France | | MP19 | | | ^3^ | |  | | |  | | | - | | |  | | | - | | |  | | |  | | | + | | - | | - | | - | | ^5,6^ | |  |
| 4 | | Coânac 1 | | France | | MP19 | | | ^3^ | |  | | |  | | | *bottii* | | | - | | | - | | | - | | | ^4,5^ | | | + | | - | | - | | - | | ^5,6^ | |  |
| 5 | | Rosières 2 | | France | | MP19 | | | ^3^ | |  | | |  | | | *bottii*, sp. | | | - | | | - | | | Fig. 4 | | | ^1,4,5^ | | | + | | - | | - | | - | | ^5^ | |  |
| 6 | | Sindou D | | France | | MP19 | | | ^3^ | |  | | |  | | | *bottii* | | | - | | | - | | | - | | | ^4,5^ | | | + | | - | | - | | - | | ^5,6^ | |  |
| 7 | | Sainte-Néboule | | France | | MP18 | | | ^3^ | |  | | |  | | | *bottii* | | | - | | | - | | | - | | | ^4,5^ | | | ?+ | | 1 il | | + | | - | | ^5,6^ | |  |
| 8 | | Monteils | | France | | MP18 | | | ^7^ | |  | | |  | | | cf. *bottii* | | | ? | | | ? | | | - | | | ^7^ | | | + | | ? | | ? | | - | | ^7^ | |  |
| 9 | | Cregols | | France | | MP18 | | | ^1^ | |  | | |  | | | sp. | | | + | | | - | | | Fig. 4 | | | ^1^ | | | - | | - | | - | | - | | ^-^ | |  |
| 10 | | Perrière | | France | | MP17b | | | ^3^ | |  | | |  | | | *bottii* | | | - | | | - | | |  | | | ^4,5^ | | | + | | 1 il | | + | | Fig. 2B | | ^5,6^ | |  |
| 11 | | Malpérié | | France | | MP17b | | | ^3^ | |  | | |  | | | ? *bottii* | | | - | | | - | | |  | | | ^4^ | | | + | | 1 hum | | - | | - | | ^5,6^ | |  |
| 12 | | Aubrelong 2 | | France | | MP17a | | | ^3^ | |  | | |  | | | ? *bottii* | | |  | | |  | | |  | | | ^4^ | | | + | | 1 svert | | - | | - | | ^5,6^ | |  |
| 13 | | La Bouffie | | France | | MP17a | | | ^3^ | |  | | |  | | | *bottii* | | |  | | |  | | |  | | | ^4^ | | | + | | 1 hum, 1psvert | | - | | Fig. 1 | | ^5,6^ | |  |
| 14 | | Lebratières 1 | | France | | MP17a | | | ^3^ | |  | | |  | | | - | | | - | | | - | | | - | | | ^-^ | | | + | | 1 svert | | - | | - | | ^5,6^ | |  |
| 15 | | Southwest Headon Hill (=Headon Hill 3) | | England | | MP17a | | | ^8^ | |  | | |  | | | *walti* | | | skull elem. | | | + | | | Figs 1, 2, 6 | | | ^8^ | | | - | | - | | - | | - | |  | |  |
|  | | |  | | England | | MP17a | | | ^9^ | |  | | |  | | | *sulcatus* | | | skull elem. | | | - | | | - | | | ^9^ | | - | | - | | - | | - | | ^-^ | |  |
| 16 | | Rodent bed, Hordle Cliff locality | | England | | MP17a | | | ^9^ | |  | | |  | | | *sulcatus* | | | - | | | - | | | Fig. 2 | | | ^9^ | | | - | | - | | - | | - | | ^-^ | |  |
|  |  | | | England | | MP17a | | | ^8^ | |  | | |  | | | *walti* | | | skull elem. | | | + | | | Figs 3-5 | | | ^8^ | | | - | | - | | - | | - | | ^-^ | |  |
| 17 | | Mammal bed, Hordle Cliff locality | | England | | MP17a | | | ^9^ | |  | | |  | | | *sulcatus* | | | skull | | | - | | | Fig. 1 | | | ^9^ | | | + | | 1 il | | + | | Fig. 1 | | ^10^ | |  |
| 18 | | Quercy Phosphorite | | France | | MP17? | | | ^11^ | |  | | |  | | | *gezei* (Mummy) | | | skull, trunk | | | - | | | + | | | ^11^ | | | - | | - | | - | | - | | ^-^ | |  |
| 19 | | Le Bretou^12^ | | France | | MP16 | | | ^3^ | |  | | |  | | | sp. | | | skull | | |  | | | Fig. 3 | | | ^1^ | | | - | | - | | - | | - | | ^-^ | |  |
| 20 | | Lavergne | | France | | MP16 | | | ^3^ | |  | | |  | | |  | | | - | | | - | | | - | | | - | | | + | | 1 VIII vert, 1 svert, 1 cor, 1 hum, 2 il | | + | | Fig. 2A, 2C-E | | ^5,6^ | |  |
| 21 | | Grisolles | | France | | MP16 | | | ^3^ | |  | | |  | | | - | | | - | | |  | | | - | | | - | | | + | | 2 hum, 3 il | | + | | Fig. 1B-D | | ^6^ | |  |
| **22** | | **Verrerie de Roches** | | Switzerland | | MP16 | | | ^13^ | |  | | |  | | | *bottii* | | | see material | | |  | | |  | | | present study | | | + | | see material | |  | |  | | present study | |  |
| **23** | | **Les Alleveys** | | Switzerland | | MP16 | | | ^14,15^ | |  | | |  | | | *bottii* | | | see material | | |  | | | **-** | | | present study | | | + | | see material | | - | | - | | present study | |  |
| **24** | | **Dielsdorf 1, 2, A, ?** | | Switzerland | | MP16-MP20 | | | ^16^ | |  | | |  | | | sp. | | | see material | | |  | | |  | | | present study | | | + | | see material | |  | |  | | present study | |  |

^1^ **Rage** **J-C. 1988.** Le gisement du Bretou (Phosphorites du Quercy, Tarn-et-Garonne, France) et sa faune de vertebres de l'Eocene superieur: I. Amphibiens et reptilies. *Palaeontographica Abt. A* **205**:3–27.

^2^ **Rage** **J-C, Roček** **Z. 2007.** A new species of *Thaumastosaurus* (Amphibia: Anura) from the Eocene of Europe. *Journal of Vertebrate Paleontology* **27 (2)**:329–336.

^3^ **Rage** **J-C. 2006.** The lower vertebrates from the Eocene and Oligocene of the phosphorites du Quercy (France): an overview. *Strata* **13**:161–173.

^4^ **Escarguel** **G, Marandat** **B, Legendre** **S. 1997.** Sur l'âge numérique des faunes de mammifères du Paléogène d'Europe occidentale, en particulier celles de l'Éocène inférieur et moyen. *Mémoires et Travaux d'école Pratique des Hautes Études, l’Institut de Montpellier* **21**:443–460.

^5^ **Roček** **Z, Lamaud** **P. 1995.** *Thaumastosaurus bottii* De Stefano, 1903, an anuran with Gondwanan affinities from the Eocene of Europe. *Journal of Vertebrate Paleontology* **15 (3)**:506–515.

^6^ **Crochet** **J-Y, Hartenberger** **J-L, Rage** **J-C, Rémy** **JA, Sigé** **B, Sudre** **J, Vianey-Liaud** **M. 1981.** Les nouvelles faunes de vertébrés antérieures à la « Grande Coupure» découvertes dans les phosphorites du Quercy. *Bulletin du Muséum National d'Histoire Naturelle, 3e Série, Science de la Terre, section C* **3 (3)**:245–266.

^7^ **Rage** **J-C. 1984.** Are the Ranidae (Anura, Amphibia) known prior to the Oligocene? *Amphibia-Reptilia* **5**:281–288.

^8^ **Maitre** **E, Hugueney** **M, Astruc** **JG, Crochet** **J-Y, Escarguel** **G, Godinot** **M, Legendre** **M, Marandat** **B, Mourer-Chauviré** **C, Rage** **J-C, Rémy** **JA, Simon-Coinçon** **R, Sudre** **J, Valette** **P, Sigé** **B. 2006.** Huit nouvelles faunes Éocènes et Oligocènes des Phosphorites du Quercy. *Strata* **13 (1)**:113–127.

^9^ **Holman** **AJ, Harrison** **DL. 1999.** *Rana* (Amphibia: Ranidae) from the upper Eocene (MP17a) Hordle cliff locality, Hampshire, England. *Palaeovertebrata* **28 (1)**:47–51.

^10^ **Holman** **AJ, Harrison** **DL. 2003.** A new helmeted frog of the genus *Thaumastosaurus* from the Eocene of England. *Acta Palaeontologica Polonica* **48 (1)**:157–160.

^11^ **Holman** **AJ, Harrison** **DL. 2002.** A New *Thaumastosaurus* (Anura: Familia Incertae Sedis) from the Late Eocene of England, with Remarks on the Taxonomic and Zoogeographic Relationships of the Genus. *Journal of Herpetology* **36 (4)**:621–626.

^12^ Probably Pelobatidae indet. and Anura indet. from the le Bretou could belong to *Thaumastosaurus* sp. and cf. *Rana* sp. (Rage 1988^1^; Figs 2,6).

^13^ **Laloy** **F, Rage** **J-C, Evans** **SE, Boistel** **R, Lenoir** **N, Laurin** **M. 2013.** A Re-Interpretation of the Eocene Anuran *Thaumastosaurus* Based on MicroCT Examination of a ‘Mummified’ Specimen. *PLoS ONE* **8 (9)**:e74874.

^14^ **Becker** **D, Rauber** **G, Scherler** **L. 2013.** New small mammal fauna of late Middle Eocene age from a fissure filling at La Verrerie de Roches (Jura, NW Switzerland). *Revue de Paléobiologie* **32 (2)**:433–446.

^15^ **Hooker** **JH, Weidmann** **M. 2000.** The Eocene mammal faunas of Mormont, Switzerland. *Schweizerische Paläontologische Abhandlungen* **120**:1–141.

^16^ **Hooker** **JJ, Weidmann** **M. 2007.** A diverse rodent fauna from the middle Bartonian (Eocene) of Les Alleveys, Switzerland: snapshot of the early theridomyid radiation. *Swiss Journal of Geosciences* **100 (3)**:469–493.
